# ‘Neurasthenia gastrica’ revisited: perceptions of nerve-gut interactions in nervous exhaustion, 1880–1920

**DOI:** 10.1080/16512235.2018.1553438

**Published:** 2018-12-25

**Authors:** Kristine Lillestøl

**Affiliations:** a Section for Medical Anthropology and Medical History, Department of Community Medicine and Global Health, Institute of Health and Society, University of Oslo, Oslo, Norway; b Department of Medical Genetics, Oslo University Hospital, Oslo, Norway

**Keywords:** Neurasthenia, neurasthenia gastrica, nervous dyspepsia, functional gastrointestinal disorders, intestinal autointoxication, brain–gut axis

## Abstract

In this paper, some of the medical literature on the historical disease-concept of ‘neurasthenia gastrica’ is reviewed. Neurasthenia gastrica was defined as a sub-unit of the wider category of neurasthenia, also referred to as nervous exhaustion or nervous weakness. Neurasthenia was a commonly used diagnostic label at the end of the nineteenth century and a few decades onwards, and was used to describe a wide variety of symptoms for which no ‘organic’ basis could be found. In neurasthenia gastrica, however, the gastrointestinal symptoms predominated, and there was considerable debate as to how the gut interacted with the central nervous system in the development of these ailments. Some of these discussions may be seen as historical precedents for the current debates on the brain–gut–microbiota axis, particularly in relation to the so-called functional gastrointestinal disorders.

Neurasthenia was a widely used diagnostic label in America and most European countries at the end of the nineteenth century. The ‘birth’ of neurasthenia as a disease category is usually dated to 1869, when two American physicians apparently independently of each other published their first works on this condition [1–3]. Of these two, Edwin van Deusen and George Miller Beard, it was the latter who became most strongly associated with the disease label (Figure 1). Beard was a New York neurologist, chiefly attending to the upper middle class patients of the city [4]. When he presented his reflections on ”Neurasthenia, or Nervous Exhaustion” for the first time, in a lecture to the New York Medical Journal Association in 1869, he opened by stating that neurasthenia, literally meaning ‘want of strength in the nerve’, was one of the most common causes and effects of disease at the time. He compared the condition to anemia, arguing that ‘Anæmia (…) is to the vascular system what neurasthenia is to the nervous. The one means want of blood; the other, want of nervous force’ [2, p. 217]. This ‘want of nervous force’ and exhaustion of the nervous system could, according to Beard, lead to the development of a vast range of symptoms, including tiredness, headaches, palpitations, anxiety, depression, and sexual impotence. The general clinical examinations did, however, rarely reveal any pathological findings [5].

**Figure 1.**
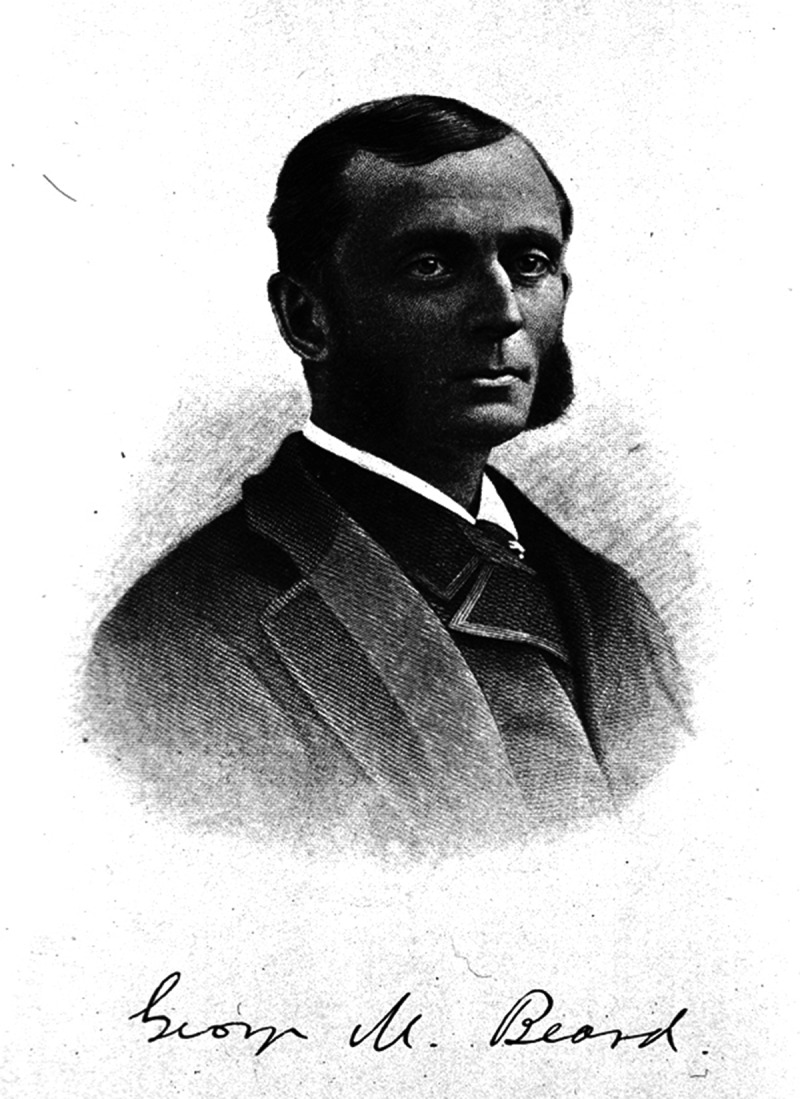
Portrait of George Miller Beard, the ‘father’ of neurasthenia. (Wikimedia Public Domain).

**Figure 2.**
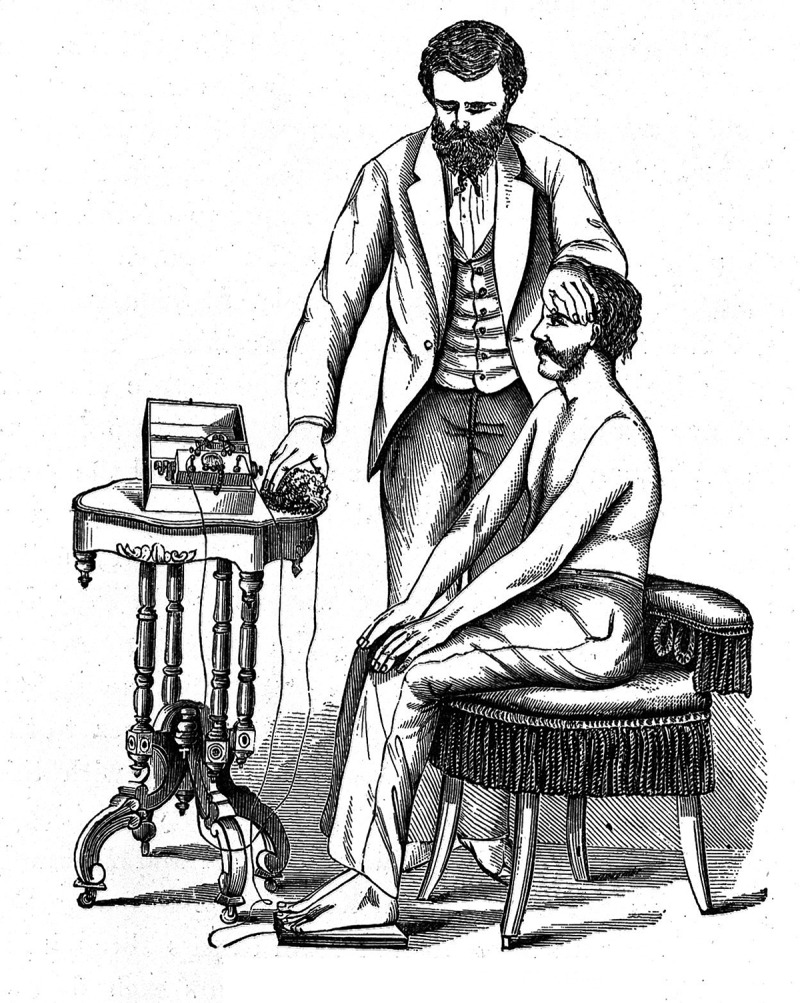
An illustration of electrotherapy (general faradization), a commonly used treatment for neurasthenia. Julius Althaus, 1873. Courtesy of the Wellcome Collection.

**Figure 3.**
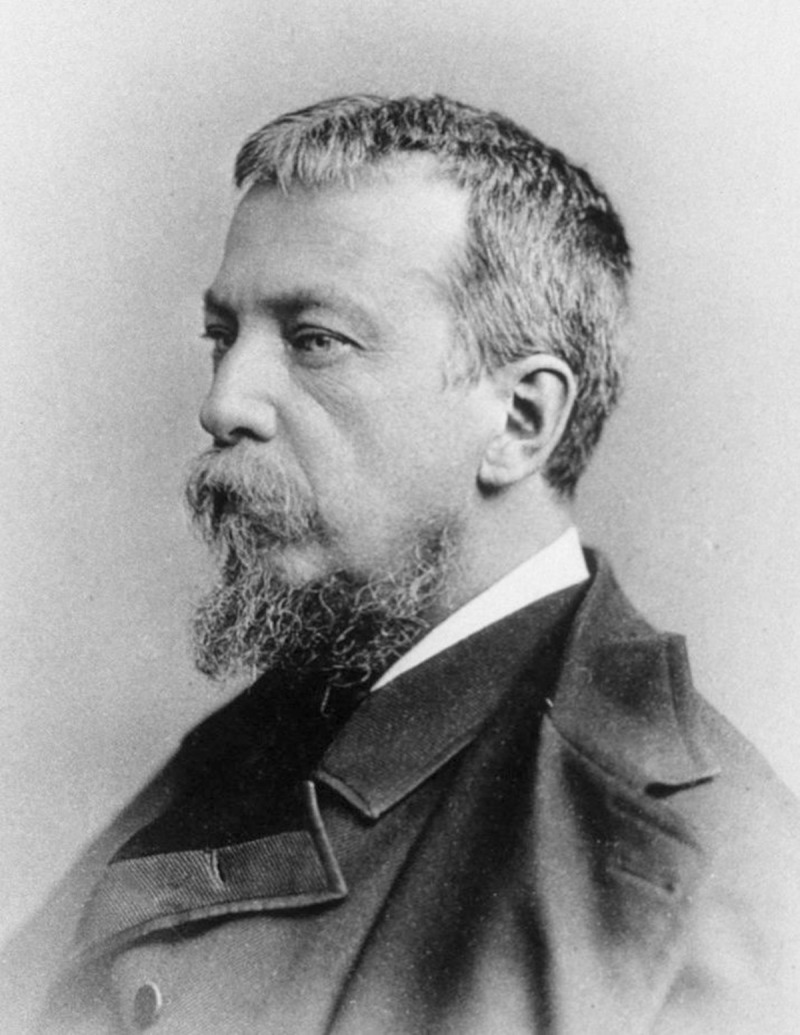
Portrait of Silas Weir Mitchell, the man behind the famous rest cure. (Wikimedia Public Domain).

When it came to etiological factors of the condition, Beard was of the opinion that ‘Neurasthenia may result from any causes that exhaust the nervous system’. Examples of such causes were a hereditary disposition, as well as ‘special exciting causes’ such as ‘the pressure of bereavement’, ‘business and family cares’, ‘sexual excesses’ and ‘the abuse of stimulants and narcotics’ [2, p. 218]. The causal explanation he became most famous for, however, was the one he presented in a later work on *American Nervousness* (1881), in which he argued that neurasthenia was to be understood as a product of modern civilization, and the rapid societal changes and hectic American life at the end of the nineteenth century [6].

The history of neurasthenia has been widely studied during the last three decades. Historians have paid particular attention to the fact that neurasthenia was interpreted differently in different cultural and national contexts [3,7,8]. The notion that neurasthenia was a product of modernization has also been widely studied [9–12]. Since the late 1980s neurasthenia has also figured in medical debates, where it has been suggested as a historical forerunner of several contested diagnoses of our time, most notably chronic fatigue syndrome (also called myalgic encephalomyelitis/ME) [13,14].

A largely neglected part of the history of neurasthenia, however, is that neurasthenia in its heyday in many cases was perceived as a disorder which was closely associated with the gut. For instance, in 1906 the Canadian physician Hugh McCallum claimed that ‘there is no known functional disease of the stomach that cannot have its cause and continuity in neurasthenia’ [15, p. 1031]. Moreover, several physicians reported that gastrointestinal complaints constituted a predominant part of the clinical picture in many neurasthenic patients, and some of these physicians felt the need to define a sub-entity of the wide neurasthenia diagnosis.

In the present paper, some of the major works on the sub-entity of neurasthenia called ‘neurasthenia gastrica’ will be reviewed. Drawing primarily on medical journals and textbooks from the German, British and American contexts during what is often described as the heyday of neurasthenia (ca 1880–1920), I explore the origins of this disease label. Moreover, I provide an overview of the clinical descriptions, causal explanations and therapeutic recommendations associated with this condition, with an overall aim of exploring how the nature of the relationship between the gut and the nervous system was perceived within the context of neurasthenia gastrica. As I will show, these historical medical discussions about possible nerve-gut interactions in neurasthenia contain several elements that may be seen as important historical precedents for the current debate about the brain–gut axis in functional gastrointestinal disorders.

## The origins of neurasthenia gastrica

Digestive symptoms played a part already in the earliest writings on American neurasthenia; George Beard as well as Edwin van Deusen mentioned dyspepsia as one of the most common complaints in their first papers on this condition [1,2]. Beard’s attention towards this part of the neurasthenic picture grew throughout the following years, and in the text that came to be the first standard work on neurasthenia, *A Practical Treatise On Nervous Exhaustion* (1880), he elaborated on the topic and described ‘Nervous Dyspepsia’ (also referred to as ‘Dyspepsie Asthénique’) as one of neurasthenia’s core characteristics [5, p. 47]. According to Beard’s experience with neurasthenic patients, nervous dyspepsia was in many cases ‘the first noticeable symptom of nervous exhaustion’, and ‘the earliest sign that the body is giving way’. The way he saw it, a functionally disordered stomach could be the only sign of neurasthenia for several years, before nervous symptoms began to develop in other parts of the body. Nevertheless, he insisted that nervous dyspepsia should be seen as a part of ‘the same general pathological condition as all the orders of symptoms here noted’, and as symptoms which might ‘follow or accompany as well as lead this multitudinous army’ of other neurasthenic symptoms [5, p. 47].

The first to coin the term neurasthenia gastrica, or gastric neurasthenia, was possibly also an American physician. In a lecture to the Rhode Island Medical Society on 15 September 1880, William F. Hutchinson presented ‘Three typical cases of neurasthenia’ [16]. In one of these cases, which was presented under the headline ‘Gastric Neurasthenia’, the patient was a 47-year-old widower from New York. Among the clinical features noted, was a ‘facial expression anxious in the extreme, with dark circles around eyes’, and his general appearance was described as ‘bad’. Moreover, he was described as ‘nervous to a distressing extent’, and as a person who ‘sheds tears upon any sudden emotion, and finds it impossible to keep still a moment’. The patient’s most bothersome complaints, however, were located to the gastrointestinal tract: ‘After drinking a large quantity of lager beer, some dozen glasses or more’, the patient had been ‘attacked with severe nausea and long continued vomiting’, which later developed into a chronic ‘congestive irritation of the entire digestive apparatus, attended by obstinate constipation.’ Hutchinson concluded that this was a ‘distinct case of nerve-exhaustion dependent upon what is actually starvation, which, however, has not produced, as yet, any appreciable organic change’ [16, p. 399–400].

The following year, an abstract of Hutchinson’s paper was presented to German medical readers through the *Schmidts Jahrbücher der in- und ausländischen gesammten Medicin* [17]. The abstract – and the disease label in particular – caught the attention of Rudolph Burkart, who at the time worked as the physician-in-charge at the water cure resort (or ‘Wasserheilanstalt’) of Marienberg. During this practice, Burkart had noticed that a large number of his neurasthenic patients presented with stomach complaints as a predominant part of their clinical picture. In 1882, he published a book in which he suggested that neurasthenia gastrica might be a useful disease label in such cases. The book was called *Zur Pathologie der Neurasthenia Gastrica (Dyspepsia nervosa)*, and as it appears, it was with this text that the concept of neurasthenia gastrica first became a topic in the European medical debate [18].

Burkart’s text was clearly inspired by Beard’s writings on neurasthenia. Beard’s monograph *A Practical Treatise On Nervous Exhaustion* had been translated into German the year before, and received much attention from members of the German medical profession [19,20]. However, Burkart’s work was also a response to the writings of one of his German colleagues, Wilhelm von Leube, who a few years before had published his first paper on nervous dyspepsia [21]. According to Leube, the symptoms of nervous dyspepsia were due to a local affection of the gut; a ‘direct mechanical irritation of over-sensitive nerves’ [22, p. 321]. Moreover, he considered nervous dyspepsia to be an independent disorder.

As pointed out by Garland, following Leube’s paper, ‘there arose an active discussion as to his assumption that the syndrome described by him was due to a local affection of the gastric nerves’ [22, p. 321]. His assumption that nervous dyspepsia should be understood as ‘eine eigenartige, isolierte Organerkrankung’ – a distinct, independent disorder – did also become a hot topic for debate in the decades to come, and in his book, Burkart made a clear stand against Leube’s views [18]. According to Burkart (and also Beard), the symptoms known as nervous dyspepsia should *not* be understood and treated as a distinct disease, but rather as a part of a general neurasthenic condition. Consequently, the label neurasthenia gastrica should, in Burkart’s opinion, merely be used as a specification in cases of neurasthenia where digestive problems (‘einer besonderen Anomalie der Magen-Darmverdaaung’) appeared to be a predominant part of the clinical picture [18].

As noted also by Arthur Bofinger, another German physician, the debate about the ‘nervous’ disorders of the gut intensified after the publication of Leube’s legendary paper ‘Über nervöse Dyspepsie’ [23]. Burkart’s description of neurasthenia gastrica rapidly received attention from his German colleagues, and the label was also taken into use in other European countries. However, neurasthenia gastrica was far from being the only suggested alternative to Leube’s nervous dyspepsia. Other labels were also proposed, and the various names express some of the subtle differences in the authors’ underlying understanding of the nature of the relationship between the disordered gut and the central nervous system [24]. For instance, as reviewed by the Norwegian physician Johan Karl Unger Vetlesen in 1886, in the European debate there was the ‘neurasthenia dyspeptica’ and also ‘neurasthenia vago-sympathicus’ suggested by the German physician Carl Anton Ewald [24]. Rossbach, on the other hand, preferred the term ‘digestive reflex neurosis’, while Rosenthal suggested ‘gastro-asthenia’ or ‘asthenic dyspepsia’. In addition, there was the ‘psychogenic dyspepsia’ preferred by Strümpell, and the ‘maladie cerebro-gastrique’ suggested by Leven [24,25]. Back on the other side of the Atlantic, Beard introduced the term ‘digestive neurasthenia’ as a new name for the clinical variety of neurasthenia previously known as nervous dyspepsia [19]. Notably, a change also occurred throughout this period with respect to the name nervous dyspepsia, which became far more widely used than Leube’s original definition had suggested. Consequently, in a number of medical texts from the early twentieth century, the two labels – nervous dyspepsia and neurasthenia gastrica – ended up being treated more or less as synonymous terms [26–29].

## Clinical descriptions

The clinical picture of gastric neurasthenia was frequently described as extremely variable, to the extent that this variability itself was said to be a characteristic of the condition [29–31]. Several authors also emphasized that the gastrointestinal symptoms of neurasthenia were in no way specific for this disorder [18,31]. Nevertheless, some symptoms seem to have been perceived as more common and typical for neurasthenia gastrica than others. Among these were a feeling of ‘fullness’ or pressure in the epigastrium (upper part of the abdomen), epigastric pain and a sense of ‘burning’ in the stomach, in addition to heartburn, nausea and ‘eructations of inodorous and tasteless gas’ [18,27,31]. These gastric symptoms were typically reported to be aggravated by intake of food [27,30], but, as a rule, they were not dependent upon the particular quality or quantity of the food ingested. As noted by – amongst others – the New York physician Anthony Bassler: ‘Sometimes the most digestible foods cause distress, while the most indigestible are borne without discomfort’ [31, p. 802]. However, other authors suggested that certain kinds of food generally did cause more trouble than others. For instance, in 1912 John Honeyford stated that ‘In most cases of gastric neurasthenia the carbohydrate portion of the food is not sufficiently acted upon’ [32, p. 17]; in other words suggesting an impaired digestion of carbohydrates. Honeyford also pointed to ‘highly seasoned dishes, smoked and cured foods, sauce and condiments’ as ‘indigestible’ foods which would aggravate symptoms [32, p. 65].

Although suggested by the name, the symptoms of neurasthenia gastrica were not limited to the stomach. Symptoms from the intestines were also reported as quite common, constipation in particular, but also sensations of ‘fulness’ or pain in different regions of the abdomen, flatulency and variable/abnormal stools. The appetite was often described as irregular [27,31,33]. Moreover, as was noted already in the works of Beard and Burkart, a number of authors also reported that the gastrointestinal complaints of neurasthenia gastrica in many cases were accompanied by symptoms apparently ‘remote’ from the digestive tract. For instance, J. Campbell McClure pointed to tiredness, inability to concentrate, capricious memory, headaches, palpitations, sleeplessness as well as vague pain in muscles and joints, as some of the most common non-gastric symptoms associated with gastric neurasthenia [34]. Robert Coleman Kemp made particular mention of ‘a sleepy feeling, or even weakness or dizziness’, and ‘marked mental depression’ as commonly associated symptoms [27, p. 382], while Charles D. Aaron reported that ‘fulness of the head, cephalalgia, migraine, inability to work, vertigo, lassitude, insomnia, hypochondriac and melancholic illusions’ were some of the most common ‘general’ symptoms of gastric neurasthenia [29, p. 355].

Despite the many symptoms, a routine physical examination of the abdomen did usually not reveal anything abnormal, and this discrepancy between the intensity of subjective symptoms and lack of pathological findings was itself considered a core characteristic of neurasthenia gastrica [18].

## The ‘nervous’ explanations

The majority of physicians who wrote about gastric neurasthenia during this period, were in accordance with Beard’s and Burkart’s understanding of neurasthenia gastrica as a symptom-complex which was part of a general neurasthenic condition, rather than a distinct, ‘local’ disorder of the gut. Consequently, the suggested disease mechanisms and medical theories related to gastric neurasthenia were to a large extent overlapping and in line with those of neurasthenia in general. In the understanding of how general neurasthenia could develop with such a wide range of symptoms, the two most essential elements were ‘loss of nerve power’ and ‘morbid exaltation of nervous sensibility’ [35, p. 45], often condensed to ‘irritable weakness’ [36, p. 2] or, in German, ‘reizbare Schwäche’ [20]. Correspondingly, the basic understanding of the disease mechanisms of gastric neurasthenia, was that the manifold symptoms were caused by an increased irritability and marked weakness of the nerves innervating the stomach [37].

As to what kind of factors that could cause such irritable weakness in the first place, there were numerous suggestions. Several authors emphasized the importance of heredity; that a nervous disposition could be inherited and congenital, and thus be a strong predisposing factor for the development of gastric neurasthenia later in life [18,26]. Examples of ‘certain conditions in the parent’ assumed to act as ‘predisposing factors in weakening the nervous system of the child’, were ‘mental and physical debility, alcoholic and sexual excesses, tubercle, syphilis, youthfulness or extreme age and neuroses of the parents’ [32, p. 9].

However, according to several physicians, the irritable weakness of the nerve-supply of the stomach might just as well develop ‘in a fit constitution after the excessive expenditure of nerve force’ [37, p. 336]. Some of the most commonly suggested factors suspected to drain the nervous system of energy, were overwork, worry, emotional excitement, ‘overstudy’ and other forms of mental over-exertion [26,27,30,37,38]. According to John Harvey Kellogg, medical doctor and superintendent of the Battle Creek Sanitarium, ‘overactivity or too prolonged activity of the brain, especially worry and harassment of the mind, unquestionably excite the abdominal brain to a harmful degree and lead to gastric and other visceral disturbance’ [39, p. 101].

So-called ‘sexual excesses’ and self-abuse (masturbation) were also common explanations, particularly in the case of male patients [26,27,30,37,38]. The British physician John Honeyford stressed the harmful effects of ‘over-indulgence in narcotic substances such as tea, coffee, tobacco &c., late hours and the want of sufficient sleep’ [32, p. 8]. Moreover, neurasthenia gastrica was observed to develop in the aftermath of other diseases, such as influenza, malaria or venereal disease [28,32,33]. Reflex irritation from other organs of the abdominal cavity – predominantly the uterus, was also described as a common cause [30].

In 1915, Captain J. Campbell McClure pointed to the ongoing war as a particularly common cause of gastric neurasthenia. As physician to the Red Cross Clinic for Physical Treatment of Officers in London, he had seen ‘several cases in which the foundation of a neurasthenia of a definitely gastric type was laid during the sieges of Ladysmith and Mafeking.’ He explained this by ‘the nerve-racking strain maintained for weeks, insufficient and coarse food, and the physical exhaustion of continued vigil’, and found it conceivable that the war would continue to ‘produce a large group of cases of this kind both in our navy and our army’ [34, p. 698].

As to how the different nerve centers could communicate with each other, and thus produce symptoms from several parts of the body when an ‘irritable weakness’ of the nervous system had developed, there were no definite answers, but several theories. George Beard, for instance, based his views on reflex theory: ‘The body is a bundle of reflex actions. An irritation in one part is liable to produce an irritation in some other part’ [19, pp. 41–42]. This was ‘true of all parts of the body’, he continued, but he singled out the stomach as one of the most important of the reflex centers. McClure pointed to the importance of the exhaustion of the vagus centres [40], while William van Valzah and J. Douglas Nisbet, on the other hand, assumed that the communication between the gut and the nervous system occurred primarily through the solar plexus:

The solar plexus, receiving all the impressions from the abdominal and thoracic organs, is very intimately associated with the cerebrum. Through it sensation, thought, and emotion influence digestion. Through it and the pneumogastric nerves digestion affects the activity of the brain. (…) It is the connecting link between the moral, the intellectual, and the vegetative life. (…) It is this highest and greatest assemblage of sympathetic centers which unites the nervous symptoms of neurasthenia gastrica. [37, p. 336]

The solar plexus was also in focus in the writings of J. H. Kellogg, who suggested there were actually two brains to be considered in neurasthenia, and they had the ability to mutually influence each other:

The region of the stomach is the seat of the solar plexus, the great abdominal brain which exercises a controlling influence over all the functions of digestion blood-circulation, elimination – all the automatic processes of animal life. The great sympathetic chain of ganglia is the center of the organic life of the body. Through the close association of the abdominal brain and the cerebrum there is an intimate connection between digestion and mental action. It is through this association of the cranial brain and the abdominal brain that mental states affect digestion so profoundly, and the reverse. [39, p. 98–99]

## Neurasthenia – primarily a disorder of the gut?

Although most authors who discussed the subject of neurasthenia gastrica perceived the gastric disorder to be a part of – or secondary to – the general neurasthenia, other physicians believed that it was the other way around, and that a disturbed gut was the primary problem in gastric as well as general neurasthenia. One of the physicians who raised criticism against the advocates for the most common understanding of the mechanisms of neurasthenia gastrica, was Thomas D. Savill, physician to the West-End Hospital for Diseases of the Nervous System in London. In his *Clinical Lectures on Neurasthenia* (1899), he accused the ‘observers of this school’ who were ‘in the habit of speaking of “gastric neurasthenia”’ for denying, or at least not adequately considering ‘the possibility of neurasthenia resulting from gastric disorder’ [41, p. 55].

Savill was, for his own part, convinced that neurasthenia in the majority of cases *was* a result of a (primary) gastric disorder, and he explained why he had come to this conclusion. After careful history-taking of 102 of his own neurasthenia patients, he had found out that as many as 74 of these patients had experienced symptoms of ‘gastric derangement’ prior to the development of other symptoms of neurasthenia. Moreover, his experience was that when patients were efficiently treated for their gastric problems, their neurasthenic symptoms faded as well.

How could this be possible? Savill’s explanation was that gastric disturbances might produce neurasthenia via an ‘autointoxic condition of the blood’, in which the ‘toxic products of digestion may have a specifically poisonous effect on nerve structures’ [41, p. 67]. He was not the only one to think along these lines. The theory of intestinal autointoxication, commonly ascribed to the French physician Charles Bouchard, was embraced by many medical doctors during the latter half of the nineteenth century, and was a commonly suggested disease mechanism for a wide range of disorders [42]. At its core was the assumption that toxic putrefactive products of digestion could cause systemic disease after being absorbed from the bowel:

The absorption of toxins from the intestinal canal – caused by changes in the digestive juices, by imperfect digestion, fermentation and by bacteria – constantly takes place, and is the chief cause in perpetuating the trouble. [43, p. 21]

The toxic products of digestion were in turn assumed to impair ‘in varying degrees the anatomical elements of the different organs and notably the nervous centres’ [36, p. 82]. Thus, by toxic attacks on the nervous system, the products of a disturbed digestive process could produce not only the local symptoms of gastric neurasthenia, such as ‘laborious digestion’, but also almost any kind of the previously mentioned ‘remote’ nervous symptoms commonly associated with the condition, such as exhaustion, headache, insomnia, palpitation and melancholia [32,36]. In 1891 the French physician Champagnac boldly claimed that autointoxication was the true ‘point of departure’ of neurasthenic disturbances [44], and two other French physicians, Gilbert Ballet and Adrien Proust, ranged it as the most important of all the modern ‘theories, which ascribe the origin of the neurasthenic conditions to disorders of the gastric functions’ [36, p. 81–82].

In neurasthenia, the process of intestinal autointoxication was often perceived as being associated with, and facilitated by, a flaccid (atonic) stomach [45]. A weakness and loss of tone in the stomach walls was assumed to lead to poor motoric function (peristalsis), with constipation as a possible consequence. This ‘intestinal stasis’ was in turn assumed to contribute greatly to an ‘imperfect’ process of putrefaction and, consequently, intestinal autointoxication. Gastric atony was also reported to be associated with a dilatation of the stomach. Moreover, gastric atony was reported to be a common characteristic of the neurasthenic stomach, in its own right.

For instance, in 1917, when describing his experiences with soldiers who had developed neurasthenia during the war, J. Campbell McClure pointed to gastric atony as a condition which at the time was ‘a source of considerable trouble to those who are dealing with war neurasthenics’. He told that during the past two years, he had met

a large number of soldiers suffering from neurasthenia, either with or without a definite history of shell shock, who, in addition to the physical exhaustion and psychasthenia common in these cases, have suffered definitely from sensations referable to the abdomen, such as aching in the left hypochondrium, pain in the epigastrium, a sensation of constriction in the lower sternal region, and a general feeling of sinking referred not only to the epigastrium but perhaps to the whole abdomen. [40, p. 600]

In addition, the soldiers frequently suffered from loss of appetite and a feeling of distention of the stomach after eating, which could persist for several hours. An X-ray examination of the stomach did, according to McClure, in the majority of cases show ‘a stomach slightly more capacious than normally and rather slow to empty’. He distinguished between two classes of such cases, with slightly different underlying mechanisms:

In the former class, who recover quickly and apparently completely after suitable treatment, I believe that the gastric atony is due to over-influence of splanchnics. (...) In the latter class, whose convalescence is long and too often incomplete, it appears likely that the nervous fault which produces the gastric atony is failure of impulses due to exhaustion of the vagus centres. [40, p. 601]

As for the actual cause of gastric atony in these cases, McClure pointed particularly to the ‘emotion of fear’:

All these men have been subjected, apart from definite shell shock, to experiences which are exhausting physically and mentally trying. (…) One has to remember in dealing with such patients at the present time that in the cases of the bravest man the emotion of fear, or if we choose to call it so, of anxiety, is a contributing factor in the production of any condition of muscular and nervous weakness. [40, p. 600]

However, as was also acknowledged by McClure, a general challenge when trying to understand the role of gastric atony in neurasthenia, was to establish whether this condition was the primary problem with neurasthenia as a secondary phenomenon, ‘or whether the gastric condition is simply the emphatic expression in the stomach of a general neurosis.’ [34, p. 697]. There were differing views. In 1903, Ballet and Proust concluded that ‘it seems certain that gastro-intestinal atony (…) [is] more often the effect than the cause of the affection’ [36, p. 3]. A few years later, however, the American surgeons MacLaren and Daugherty argued that gastric atony was ‘the original cause of neurasthenia’ [46].

Another suggested characteristic of the gut in gastric as well as general neurasthenia, was the so-called ptosis (sagging, downward displacement) of the stomach and/or intestines and other organs of the abdominal cavity, referred to as gastroptosis, enteroptosis, and visceroptosis, respectively. Ptosis could be associated with gastric atony and was also described as a possible facilitator for intestinal autointoxication; the latter through ‘stagnation’ of the contents of the stomach and intestines. It was also assumed that the descended organs could produce changes in the circulation of the various viscera of the abdominal cavity, with unfortunate consequences [38].

The physician who was usually credited for having been the first to describe a possible causal association between such ”sinking of the viscera” and neurasthenia in the early 1880s, was the French physician Glénard [24,47,48]. During the following decades, several other physicians reported to have observed this abnormality in a number of neurasthenic patients, and some of them even went so far as to call it a ‘stigma neurasthenicum’ – a distinct sign of neurasthenia.

One of them was the Canadian physician Hugh McCallum, who in 1906 stated that he looked upon ‘ptosis of any of the abdominal viscera as a stigma of neurasthenia, and quite as pathognomonic of it as a sharp haemoptysis is of pulmonary tuberculosis’ [15, p.1032]. In a previous paper, he stated that as many as 90 percent of female cases of neurasthenia were ‘victims of visceroptosis’, and he argued that the symptoms of visceroptosis were practically the same as those of neurasthenia – ‘with or without local distress’, which suggested a direct causal link [49]. Examples of symptoms of gastric neurasthenia which were also reported in visceroptosis, were a disturbed appetite, ”a sense of fullness in the epigastrium, belching, acid taste” and burning pain in the epigastrium after eating. The general nervous symptoms included ‘general weakness, changeable and depressed moods, headaches and fulness of the head, vertigo, (…) disturbance of sleep’, and a number of other ailments [38, p. 540]. Among the etiological explanations for the visceroptosis as such, were a ‘bad standing posture’, ‘badly-fitting garments’, high-heeled shoes and corsets, ‘the imperfect use of the lower zone of the thorax, the absence of fat, and the want of tonicity in the abdominal musculature leading to defective intra-abdominal pressure’ [47,49, p. 345, 50].

This enthusiasm for ptosis as a potential direct causal explanation in gastric neurasthenia was, however, not shared by every author on the subject. McCallum was instantly criticized for having ‘magnified’ the importance of visceroptosis in neurasthenics [15], and several authors argued that although enteroptosis certainly did exist in a number of neurasthenic patients, it was the exception rather than the rule [36,48]. In his thesis on neurasthenia gastrica published in 1912, John Honeyford stated that Glenard’s conclusion ‘that enteroptosis was a causative factor in the establishment of neurasthenia’ had been ‘shown to be erroneous as no symptoms of neurasthenia have been detected in many cases where both gastroptosis and enteroptosis were present.’ He concluded that instead of viewing ptosis of the viscera as the primary cause of neurasthenia, ‘the consensus of present day opinion favours the idea that gastroptosis is the result or concomitant of neurasthenia, and that once set up it frequently establishes symptoms of its own’ [32, p. 4–5]. In other words, as in the case of gastric atony and other alleged abormalities of the neurasthenic gut, the nature and directionality of the relationship between these gastrointestinal conditions and the symptoms of gastric and general neurasthenia were in no way clear-cut matters. In 1903, Ballet and Proust made the following summary of the situation:

In short, the relations between dyspeptic states and neurasthenia may, we think, be summed up as follows: in the majority of patients suffering from nervous exhaustion the dyspepsia has merely the value of a symptom, but of an important symptom, since it may contribute largely to keeping up the neuropathic state. In certain cases – sufficiently numerous it seems – the disorder of the digestive functions has been the primary cause of neurasthenia; and it is against it that the treatment must principally be directed. [36, p. 85]

## Treatment for the nervous gut

The main principles for treatment of neurasthenia in general, and also for neurasthenia gastrica, were removal of the eliciting causes (when possible), and restoration of the nervous energy. This could be done in a number of ways. In the many cases when overwork, daily worries and other kinds of mental strain were suspected as the main causes of the nervous weakness, one way of achieving both these goals, was to ‘take a cure’ or in other ways remove oneself from one’s customary surroundings in order to rest, preferrably at a quiet retreat. According to Hemmeter, this was particularly important for ‘American business men, who, with admirable energy but with little regard for their own health, persist in executing work which is too severe for their mental and physical constitution’. These men, he continued, ‘must be taught that the prime factor in successful treatment is rest, *rest*, REST!’ [30, p. 765]

Rest and isolation were, however, controversial modes of treatment, and several authors warned against exaggerations in this respect. Thus, exercise in suitable amounts, preferably outdoors, was frequently recommended as part of the therapeutic regime: ‘Horseback riding, golf, yachting, fishing, shooting, camp life for a few weeks, a pleasure trip, all give excellent results’ [27, p. 383]. A more passive form of physical stimulation was also often encouraged: ‘There is no doubt that massage improves the nutrition of the muscles and nerves, and favors a vigorous circulation, metabolism, and regular evacuation’ [30, p. 766].

A more literal way of ‘recharging’ the nervous system could be performed through electrotherapy (Figure 2). According to George Beard, electricity was one of ‘the very best’ remedies for the nervous dyspepsia associated with neurasthenia [5]. The therapy could take many forms; it could be general and directed towards the whole nervous system, or it could be more locally targeted. One example of the latter, in the therapeutic recommendations for neurasthenia gastrica, was described by W. Fenwick:

For the stomach a constant current of 3 to 5 milliampères is passed through the epigastric for twenty minutes daily, the negative electrode being applied over the lower dorsal region and the positive one immediately below the left costal margin. [28, p. 233]

Other authors preferred ‘direct electrisation of the organ by means of a metallic wire inserted into the ordinary stomach-tube’, but Fenwick found this procedure ‘unpleasant to the patient and tedious of application’. Electricity could also be used as a remedy specifically targeted to relieve constipation, based on the following procedure: ‘One pole is inserted into the rectum and the other, consisting of a large metal disc, is successively applied to the surface of the abdomen at different points along the course of the large intestine. The interrupted current is to be preferred to the constant one, and each sitting should last for about half an hour’ [28, p. 233].

The dietary advice given to patients with gastric neurasthenia varied to a great extent. As pointed out by Franz Riegel: ‘There is no particular diet for these cases. The patients must be taught what to eat and how to nourish themselves. A strengthening diet should always be given, and an irritating diet should be avoided’ [26, p. 813]. Kemp argued along the same lines: ‘The diet should be abundant, the patient avoiding highly seasoned food, alcohol, strong coffee, and excessive smoking’ [27, p. 383]. Honeyford recommended a decreased intake of carbohydrates and an increased amount of proteins (fresh meat) [32]. Occasionally a ‘fattening cure’ was prescribed, particularly in severe cases associated with anorexia and weight loss [26, p. 813]. A general advice given with respect to the intake of food, was that ‘In every instance mastication must be thoroughly performed, a sufficient time be allowed for each meal, and no exercise permitted for an hour afterward.’ [28, p. 234].

Certain drugs were assumed to be strengthening and to exert a ‘tonic’ effect on the nervous system, such as arsenic and strychnine, while bromides were believed to lessen the nervous symptoms and improve sleep [27, p. 384]. Moreover, several authors, such as Riegel, emphasized the importance of so-called psychic treatment in neurasthenia gastrica:

Psychic treatment is still more important than all these methods, for the personal influence of the physician is of fundamental importance in the treatment of these cases. Only if the patient has full confidence in the physician can we expect any good results. [26, p. 813]

Hugh MacCallum, too, stressed the importance of the ‘training of the mind’ for the patients. He had experienced that this might be helped by the reading of certain books: ‘It has become my practice to reach certain patients by way of the printed page after failing with oral instruction. Often a passage from the Bible is more impressive than volumes of secular literature.’ [15, p. 1032].

One particularly popular therapeutic regime for neurasthenics in general, which included most of these elements to a smaller or lesser extent, was the so-called ‘rest cure’ developed by the American neurologist Silas Weir-Mitchell (Figure 3) [3, pp. 25–35]. The cure typically lasted from six to eight weeks. Strict bed rest and isolation from family and friends were some of the key elements of the regime, in addition to overfeeding (a fatty diet mainly based on large quantities of milk), massage and electrotherapy. The cure also had a moral element, and the personal qualities of the doctors and nurses in charge were important for a successful result [51].

Weir-Mitchell’s rest cure was also recommended for sufferers from gastric neurasthenia, and in cases of neurasthenia gastrica associated with visceroptosis, the Weir-Mitchell cure was described as ‘the only proper treatment’ [52]. However, several authors argued that certain modifications of some of the elements had to be made. For instance, in many cases of gastric neurasthenia the long-lasting immobilization usually included in the Weir-Mitchell cure was considered to be harmful; particularly in those cases where gastric atony was assumed to be a part of the clinical picture. In these cases, immobility was thought to make matters worse and increase the gastrointestinal atony and associated constipation, and a ‘partial rest cure’, with massage, ‘passive movements’ and faradic electricity was recommended instead [32,36]. The element of overfeeding in Mitchell’s cure was another topic for debate when it came to the gastric form of neurasthenia. As pointed out by McClure: ‘One has to remember that in all these cases there is present a stomach which has lost its tone in greater or lesser degree, which is unusually capacious, and which is very slow to empty. Rest in bed and over-feeding will not help this local condition’ [34, p. 698].

In those cases where some kind of abnormality of the gastrointestinal tract was understood as the cause of gastric (and general) neurasthenia, the therapeutic advice given was somewhat different. For instance, when intestinal autointoxication was considered to be the primary problem, the aim of the treatment was to remove the source of toxemia. This could be done by improving the ‘elimination’ and reducing the often-associated constipation, by the means of drugs (‘emesis or lavage’) or so-called ‘colonic flushings’ [53]. Moreover, yoghurt was assumed to inhibit the toxic putrefactive processes of the intestines [54]. In some cases, a more radical and invasive mode of treatment option was suggested. As pointed out by Campbell McClure:

In a certain small proportion of cases it may be even necessary, on account of long-continued and severe gastro-intestinal toxæmia which resists any other form of treatment, to remove the colon and implant the ileum into the sigmoid, as recommended by Sir Arbuthnot Lane. [34, p. 699]

Sir Arbuthnot Lane was a British surgeon who became particularly associated with surgical treatment of intestinal autointoxication (‘alimentary toxæmia’), and he also recommended this treatment for neurasthenics [55]. However, McClure emphasized that such operations should not be undertaken lightly; they should ‘not be resorted to until every other known means of treatment of these cases has been proved, after careful and long-continued trial, to be a failure.’ [34, p. 699]

Surgery was also sometimes recommended in cases where gastric atony or ptosis of one of the abdominal organs was perceived as a main cause of the neurasthenic symptoms [47,56]. For instance, the American surgeon John F. Sheldon argued that in cases where the neurasthenia could be seen as secondary to gastric atony and associated complications, a ‘gastro-enterostomy, with closure of the pylorus’ would give the patients ‘complete and permanent relief, not only from the stomach symptoms, but also from the neurasthenia and constipation’ [57, p. 36]. In the cases of ptosis, George N. Kreider was of the opinion that ‘hundreds – yes, thousands – of women have been condemned to a miserable existence as hysterics or neurasthenics, who could be relieved if their abdominal ptosis were considered and relieved by bandages or operation’ [47, p. 2036]. Other physicians were far more critical and warned strongly against the use of surgical treatment in such cases, arguing that ‘no operation will take away the muscular atony but will rather aggravate it’ [46, p. 310].

The general impression left by the majority of physicians who were engaged in the medical debate about neurasthenia gastrica is that this was a challenging condition which was hard to combat. The prognosis was frequently described as poor, in the sense that the condition would often become chronic and relapse after brief intermissions of improvement. As summed up by Riegel, it was considered ‘impossible to formulate any general rules; the only way to treat these cases correctly is to individualize and to weigh carefully all the conditions in each case’ [26, p. 813].

## The end of neurasthenia gastrica?

In summary, the present study shows that neurasthenia gastrica as it was perceived by Western physicians around 1900, was a many-faceted condition and disease concept. The clinical picture was characterized as highly variable, and although gastrointestinal complaints were presented as the core manifestations of the condition, the definitions also included ‘remote’ nervous symptoms, such as fatigue, anxiety and depression. In this respect, many of the clinical descriptions of gastric neurasthenia show a great resemblance to those of the conditions we now know under the name of functional gastrointestinal disorders [58–61]. Parallels between the past and the present may also be drawn when it comes to some of the possible causes for ‘nervous’ disorders of the gut; infectious disease, emotional/mental strain and hereditary factors are among the suggested contributing factors in the development of functional gastrointestinal disorders today, as they were for neurasthenia gastrica more than a hundred years ago [60].

The historical texts studied in the present paper also clearly show that the physicians who dealt with neurasthenia gastrica around 1900 raised and struggled with many of the same questions as clinicians and researchers do today, when it comes to trying to understand the true nature of the pathways of communication between the gut and the central nervous system in functional gastrointestinal disorders. Although our current concept of a brain–gut axis was not explicitly used in the writings on neurasthenia gastrica, the reasoning around these issues nevertheless went along some of the same lines then as it does today, although in different shapes. For instance, one interesting parallel is the understanding of the vagus nerve as having a crucial role in the brain–gut communication; a theory which is acknowledged today [62,63], which was also (although more vaguely) suggested by some of the authors writing about neurasthenia gastrica. Moreover, the notion that we have an ‘abdominal brain’ which is interacting with our other brain, as was suggested in the debate about neurasthenia gastrica, would fit very well with the language in the current debate, where the enteric nervous system is frequently referred to as our ‘second brain’ [64]. Intriguingly, with the causal theory of intestinal autointoxication, the history of gastric neurasthenia also contains an element which may be seen as a historical forerunner of the present-day interest in the potential role of the microbes of the gut in the development of functional gastrointestinal (and other) disorders (the microbiome-gut-brain axis) [62,63].

It has been stated by many that the ‘golden age’ of the diagnosis of neurasthenia ended around 1920 in America and most European countries [7,13]. Apparently, and probably as a consequence, this was also the case for the label neurasthenia gastrica. Nevertheless, the debates surrounding this historical condition are still highly relevant, and should serve as an important backdrop for our current attempts to reach a more complete understanding of how the brain, gut and microbiota interact in (gut) health and disease.
